# Resistance Training in Spontaneously Hypertensive Rats with Severe Hypertension

**DOI:** 10.5935/abc.20160019

**Published:** 2016-03

**Authors:** Rodrigo Vanerson Passos Neves, Michel Kendy Souza, Clévia Santos Passos, Reury Frank Pereira Bacurau, Herbert Gustavo Simoes, Jonato Prestes, Mirian Aparecida Boim, Niels Olsen Saraiva Câmara, Maria do Carmo Pinho Franco, Milton Rocha Moraes

**Affiliations:** 1 Programa de Pós-Graduação em Educação Física, Universidade Católica de Brasília, Brasília, DF - Brazil; 2 Programa de Pós-Graduação em Medicina Translacional, Universidade Federal de São Paulo, São Paulo, SP - Brazil; 3 Laboratório de Imunologia Clínica e Experimental, Divisão de Nefrologia, Universidade Federal de São Paulo, São Paulo, SP - Brazil; 4 Divisão de Nefrologia, Departamento de Medicina, Universidade Federal de São Paulo, São Paulo, SP - Brazil; 5 Escola de Artes, Ciências e Humanidades, Universidade de São Paulo, São Paulo, SP - Brazil; 6 Instituto de Ciências Biomédicas, Universidade de São Paulo, São Paulo, SP - Brazil; 7 Departamento de Fisiologia, Universidade Federal de São Paulo, São Paulo, SP - Brazil

**Keywords:** Hypertension, Strength Muscular, Resistance Exercise, Animal model

## Abstract

**Background:**

Resistance training (RT) has been recommended as a non-pharmacological treatment for moderate hypertension. In spite of the important role of exercise intensity on training prescription, there is still no data regarding the effects of RT intensity on severe hypertension (SH).

**Objective:**

This study examined the effects of two RT protocols (vertical ladder climbing), performed at different overloads of maximal weight carried (MWC), on blood pressure (BP) and muscle strength of spontaneously hypertensive rats (SHR) with SH.

**Methods:**

Fifteen male SHR [206 ± 10 mmHg of systolic BP (SBP)] and five Wistar Kyoto rats (WKY; 119 ± 10 mmHg of SBP) were divided into 4 groups: sedentary (SED-WKY) and SHR (SED-SHR); RT1-SHR training relative to body weight (~40% of MWC); and RT2-SHR training relative to MWC test (~70% of MWC). Systolic BP and heart rate (HR) were measured weekly using the tail-cuff method. The progression of muscle strength was determined once every fifteen days. The RT consisted of 3 weekly sessions on non-consecutive days for 12-weeks.

**Results:**

Both RT protocols prevented the increase in SBP (delta - 5 and -7 mmHg, respectively; p > 0.05), whereas SBP of the SED-SHR group increased by 19 mmHg (p < 0.05). There was a decrease in HR only for the RT1 group (p < 0.05). There was a higher increase in strength in the RT2 (140%; p < 0.05) group as compared with RT1 (11%; p > 0.05).

**Conclusions:**

Our data indicated that both RT protocols were effective in preventing chronic elevation of SBP in SH. Additionally, a higher RT overload induced a greater increase in muscle strength.

## Introduction

Hypertension is well known as one of the main chronic diseases affecting modern society.^1^ It is highly prevalent worldwide and is considered a major risk factor for increased mortality.^2^ The progressive increase in BP may result in severe hypertension (SH), with systolic BP (SBP) reaching values over 180 mmHg, leading to subsequent end-organ damage, elevated arterial stiffness and left ventricular hypertrophy.^1,3^ Among the treatment methods, physical exercise is considered an interesting non-pharmacological adjunct to conventional therapy because of its efficacy and low cost, with minimal side effects if prescribed properly.^4^

The antihypertensive effects of resistance training (RT) in individuals with hypertension are less studied, with most of these studies being conducted in medicated hypertensive individuals.^5^ Yet, our studies showed the beneficial effects of RT on muscle strength, body composition and blood pressure (BP) in non-medicated hypertensive stage-1 patients.^6,7^ Other studies with RT evidenced reductions in cardiovascular risk factors,^8^ including a lower cardiovascular overload during physical activities.^9^ In turn, muscle strength is also directly associated with lower mortality in hypertensive patients.^10^

Of note, a reduced number of studies investigated the effects of the aerobic exercise (AE) intensity on individuals with SH at a high risk of mortality.^11,12^ We have demonstrated that AE intensity influences both nitric oxide release and post-exercise BP reduction in hypertensive women.^13^ However, the effect of the RT intensity has been less studied.^5^ Although RT at higher intensity leads to greater neuromuscular adaptations, such as increased strength and muscle hypertrophy,^5,6^ which are important for health and quality of life,^10^ there is a lack of data in literature regarding the role of the RT intensity on BP control.

Moreover, there is no consensus regarding the dose-response of RT intensity on BP of humans with SH.^4,5^ Thus, the resistance exercise mode and intensity to be tolerated by patients with hypertension that would optimize the hemodynamic benefits while avoiding musculoskeletal injuries and acute cardiac complications still remain to be determined.^6,7,14^ Yet, there is no study investigating the effects of different intensities of RT in BP control for individuals with SH.^5^

Spontaneously hypertensive rat (SHR) - a polygenic animal model for essential hypertension,^3^ has been widely used to investigate the effects of AE on BP control.^15-18^ They are normotensives at birth and become hypertensive throughout life, like some humans. Without treatment, these animals will develop SH.^3^ However, studies regarding resistance exercise in SHR were conducted only under acute interventions.^19,20^

Thus, the present study was designed to investigate the effects of two RT protocols, one prescribed relative to body weight (BW),^21^ and the other based on the maximal weight carried test (MWC)^22^ performed at different intensities, on BP and muscle strength in hypertensive rats with SH. We hypothesize that a higher intensity RT may be safe and would be more effective in reducing BP and increasing muscle strength in animals with SH.

## Methods

### Animals

All the procedures were approved by the Institutional Ethics Committee on Animal Use, Federal University of São Paulo-UNIFESP (CEUA: 922985/2014).

Five male Wistar-Kyoto (WKY) rats and fifteen SHR rats with 17 weeks of age were obtained from the CEDEME/UNIFESP. The animals were housed in collective cages (5 animals/cage) and were maintained at a 12-12h dark-light cycle at 22 ± 2°C and 55 ± 10% relative humidity, and fed standard chow (Nuvital^®^ CR1, Sao Paulo, Brazil), receiving water *ad libitum*. The BP values of the SHR groups start to increase after the fourth week of life, and from the fifth to the seventh week hypertension is installed. From this period, if left untreated, these animals will develop SH - SBP ≥ 180 mmHg, according to the VI Brazilian Guidelines on Hypertension.^23^ This shows that, following 12 weeks of training (excluding the two weeks of training adaptation), the age of the studied animals was 31 weeks at the end of the intervention.

### Experimental Groups

The animals were divided into four groups: sedentary WKY rats (SED-WKY), sedentary SHR (SED-SHR), SHR RT relative to BW (RT1) and SHR RT based on MWC (RT2). The animals in the trained groups completed 3 weekly sessions of RT for 12 weeks between 06:00 and 08:00 p.m. The SED groups were kept in a box with the same dimensions of the training apparatus for 10 min to simulate the stress of handling and the environmental conditions experienced by the trained groups.

### Familiarization with the Vertical Ladder

**Figure 1:**
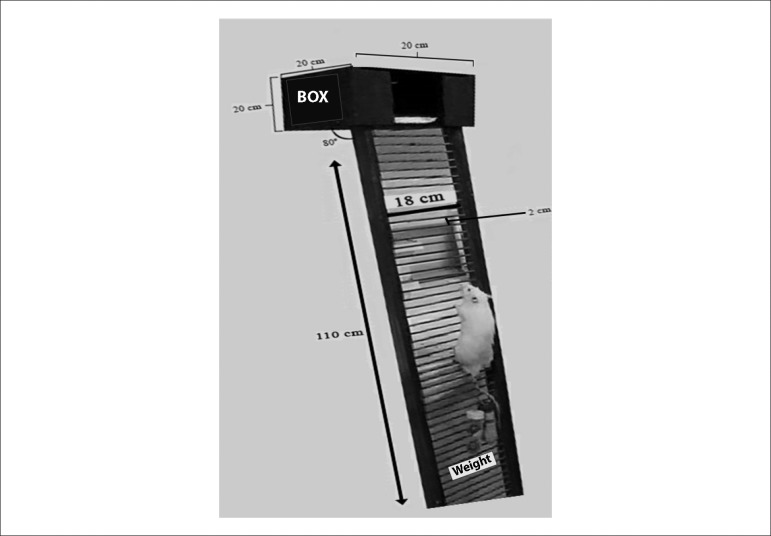
Apparatus used to perform resistance training in the rats, adapted from Cassilhas et al. 2012. Ladder 110cm high, 18 cm wide, 2 cm between grid steps and 80° incline. Box (L × W × H = 20 × 20 × 20 cm) located centrally at the top of the ladder served as a shelter during the resting period for the exercising rats.

Initially, all rats were adapted to the RT protocol by climbing a vertical ladder (110 cm high•18 cm wide, 2 cm grid, 80° incline) Figure 1. A housing chamber (L•W•H = 20•20•20 cm) was located at the top of the ladder and served as a shelter during the resting period. The familiarization consisted of climbing the ladder with the load apparatus without weight for two consecutive weeks, three times per week every other day with a total of six sessions for adaptation as has already been described.^22^

### BP Measurement

**Figure 2:**
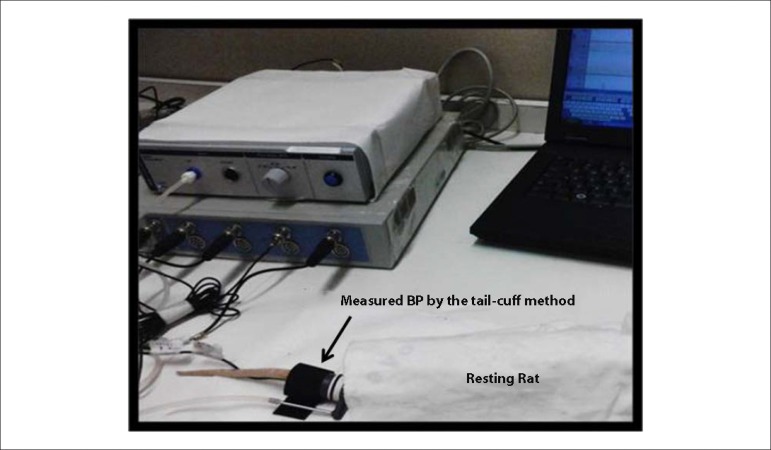
Blood pressure measured by the tail-cuff method with the rats under conscious condition.

The SBP was measured using the tail-cuff method with the rats under conscious condition with PowerLab system (ADInstruments, Inc., Sydney, Australia). This tail-cuff method (Figure 2) is a sensitive and accurate approach for the noninvasive measurement of BP in conscious SHR.^24^ SBP was measured once a week at the same time each day (between 6:00- 8:00 p.m.) to allow the animals to become adapted to the procedure.^25^ The rate-pressure product (RPP) was calculated as the product of HR and SBP. SBP, HR and BW measurements were taken on a weekly basis by the same evaluator.

### Maximal Weight Carried Test (MWC)

Two days after the familiarization procedure, all animals of the training groups had their MWC determined. For the initial climb, the weight carried was 75% of the animal's BW. After this, an additional 30g of load was added, until a maximal load was reached when the rat could not climb the entire length of the ladder between 4-9 attempts. Failure was determined when the animal could not progress up the ladder after three consecutive stimuli in the tail (using tweezers), with a 60-s rest period between each climb. The heaviest load that the animal successfully carried over the entire length of the ladder was considered the rat's MWC for that test session. Then, the next test session consisted of a ladder climb with 50%, 75%, 90%, and 100% of the rat's previous MWC with a rest interval of 60 seconds between each climb. For the subsequent ladder climbs, a 30-g load was added until a new MWC was determined; the recovery period between each climb was 120 s.^22^ This procedure was applied in the first week and repeated every 15 days throughout the 12 weeks in both groups (RT1 and RT2) in order to determine the time-course adaptations of muscle strength and the prescription of the RT2 group training intensity.

### RT Protocols

Following the MWC, both RT groups (RT1 and RT2) completed three sessions / week in non-consecutive days, between 6:00 and 8:00 p.m. for 12 weeks, totalizing 36 sessions consisting of 6-8 climbing sets of 10-12 repetitions, 1' pause between sets, with a mean duration of each training session of ~10-12 minutes. The load adjustments were performed every 15 days according to the animal's BW or the MWC test. The relative intensity of each training protocol is described in Table 1.

**Table 1:** Progression of the intensity and volume of training loads for both protocols of resistance training

**Training week**	**Ladder climbs**	**Relative to the MWC**		**Relative to the BW**		**RT1 Total overload lifted (g)**	**RT2 Total overload lifted (g)**
**% Load RT1**	**% Load RT2**	**% Load RT1**	**% Load RT2**
1^st^	1 to 3	20	30	30	51	2226±100	2390 ± 94*
	4 to 6	33	50	50	84		
2^nd^ - 3^rd^	1 to 2	20	30	30	51	6014 ± 54	7745 ± 969*
	3 to 6	33	50	50	84		
	7	40	60	60	101		
4^th^ - 12^th^	1 to 2	20	30	30	51	37853 ± 88	74164 ± 1366*
	3 to 4	33	50	50	84		
	5 to 6	40	60	60	101		
	7 to 8	46	70	70	118		

Modified from Cassilhas et al.^21^ MWC: maximal weight carried; BW: body weight; Total overload = sets•repetitions•weight. All values are presented as means ± SD.

* p < 0.05 vs. RT1.

RT1 Protocol: This protocol used the animal's BW to determine the intensity of the RT sessions. A progressively heavier load using conical tubes of 50 mL with weights inside and fixed to the proximal part of the animal's tail with a Coastlock Snap Swivel and Scotch Rubber Tape (Scotch 3 M, Sao Paulo, Brazil) was used as described by Cassilhas et al.^21^ RT2 Protocol: Rat's MWC test was used to calculate and prescribe intensity for RT; this protocol was adapted from Hornberger and Farrar.^22^

### Tissue Collection

Forty-eight hours after the last training session, the rats were euthanized by decapitation. The gastrocnemius and soleus muscles were removed and weighed immediately.^22^ Gastrocnemius was chosen because of its greater proportion of type-II muscle fibers, while soleus presents a higher amount of type-I fibers. Moreover, these muscles present almost all fibers across the middle belly of the muscle and are distributed from tendon to tendon.^8^

### Statistical Analysis

All results are expressed as means ± standard deviation of the mean (SD). To compare BP, strength gains, sum of all weight lifted, and the animal's BW values within and between sessions Split plot ANOVA (mixed ANOVA) with *post hoc* Bonferroni was used and the level of significance was p < 0.05.Statistical analysis was performed using the GraphPad Prism 6.0 software (GraphPad Software, Inc, CA, USA).

## Results

### Body and Muscle Weights

BW and wet weight of the gastrocnemius and soleus are presented in Table 2. Pre and post-training BW within all groups were significantly different (p < 0.05). There was no significant difference in gastrocnemius and soleus muscle weight (p > 0.05). Therefore it was not need to normalize muscle mass for differences in BW.

**Table 2:** Anthropometric and hemodynamic data for the WKY and SHR rats pre and post-resistance training

	**SED-WKY (n = 5)**		**SED-SHR (n = 5)**		**RT1-SHR (n = 5)**		**RT2-SHR (n = 5)**	
	**Pre**	**Post**	**Pre**	**Post**	**Pre**	**Post**	**Pre**	**Post**
BW (g)	268 ± 32	321 ± 18^a^	330 ± 9^b^	355 ± 11^a^^,^^b^	309 ± 14^b^^,^^c^	342 ± 23^a^^,^^b^	324 ± 24^b^	345 ± 21^a^^,^^b^
MW (g)	------	1.77 ± 0.15	------	1.77 ± 0.16	------	1.84 ± 0.11	------	1.83 ± 0.05
SW (g)	------	0.13 ± 0.02	------	0.14 ± 0.02	------	0.13 ± 0.02	------	0.14 ± 0.03
GW (g)	------	1.64 ± 0.14	------	1.63 ± 0.15	------	1.71 ± 0.11	------	1.69 ± 0.04
SBP (mmHg)	119 ± 4	130 ± 6^a^	206 ± 10^b^	225 ± 7^a^^,^^b^	199 ± 6^b^	194 ± 6^b^^,^^c^	206 ± 13^b^	199 ± 8^b^^,^^c^
HR (bpm)	343 ± 28	377 ± 42	426 ± 30^b^	435 ± 55	482 ± 15^b^	430 ± 11^a^	445 ± 27^b^	407 ± 50
RPP (mmHg•bpm)/100	408 ± 42	490 ± 41^a^	877 ± 80^b^	979 ± 134^b^	959 ± 41^b^	834 ± 28^a^^,^^b^	917 ± 26^b^	810 ± 101^b^^,^^c^

WKY: Wistar Kyoto Rat; SHR: Spontaneously Hypertensive Rat; SED: sedentary; BW: body weight; MW: muscle weights (gastrocnemius+soleus); SW: soleus weight; GW: gastrocnemius weight; SBP: systolic blood pressure; HR: heart rate; RPP: rate-pressure product. The values were compared within each group and between groups.

a p < 0.05 vs PRE;

b p < 0.05 vs SED-WKY;

c p < 0.05 vs SED-SHR. Data are presented as mean ± SD.

### Cardiovascular Changes

The reproductibility of SBP measures was assessed by Pearson's coefficient of variation of BP data, which demonstrated a good reliability of BP data over the 12-week experimental period, SED-WKY 2 ± 1%, SED-SHR 1 ± 1%, RT1-SHR 3 ± 1% and RT2-SHR 2 ± 1%. The results of cardiovascular parameters are presented in Table 2. The baseline SBP of the SHR groups (206 ± 10, 199 ± 6, and 206 ± 13 mmHg, SED-SHR, RT1, and RT2 - respectively) were higher as compared with those of the SED-WKY group (119 ± 4 mmHg - p < 0.05). After twelve weeks, SBP of the SED-SHR increased by 9% (∆ = 19 mmHg, p < 0.05) as compared with baseline, while SHR RT1 and RT2 groups presented a decrease by 2.5% (∆ = -5 mmHg; p > 0.05) and 3.4% (∆ = -7 mmHg; p > 0.05) in BP at the end of training, respectively.

There was a decrease in HR for the group RT1 post-training (482 ± 15 *vs*. 430 ± 11 bpm; p < 0.05). In addition, there was no significant difference in HR in the higher-intensity RT2 group (445 ± 27 *vs*. 407 ± 50; p > 0.05). The baseline RPP of the hypertensive rats (SED-SHR, RT1, and RT2) assessed throughout the training was higher when compared with that of the normotensive rats (SED-WKY, p < 0.05). The RT1 group presented a decrease in RPP pre vs. post-training (959 ± 41 vs. 834 ± 28 (mmHg•bpm)/100; p < 0.05), while there was no significant difference for the RT2 group on RPP pre vs. post-training (917 ± 26 vs 810 ± 101 (mmHg•bpm)/100; p > 0.05).

### Time course BP

**Figure 3:**
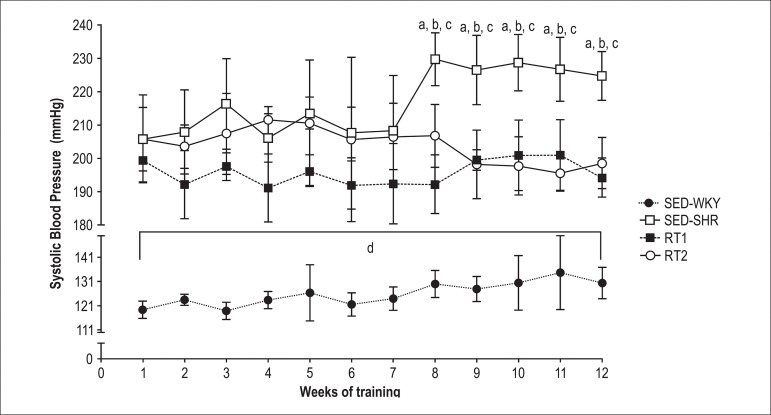
Behavior of systolic blood pressure in WKY rats and SHR. a, p < 0.05 vs. pre (1^st^ week); b, p < 0.05 vs. RT1; c, p < 0.05 vs. RT2; d, p < 0.05 vs. SHR groups. All values are presented as means ± SD.

There was no difference in SBP within groups (pre-training vs post; p > 0.05; Figure 3), except in the SED-SHR group (p < 0.05). The SBP in the SED-SHR group increased at week 8 of protocol as compared with the trained groups; this response remained until the end of the study (p < 0.05).

### Muscle Strength

**Figure 4:**
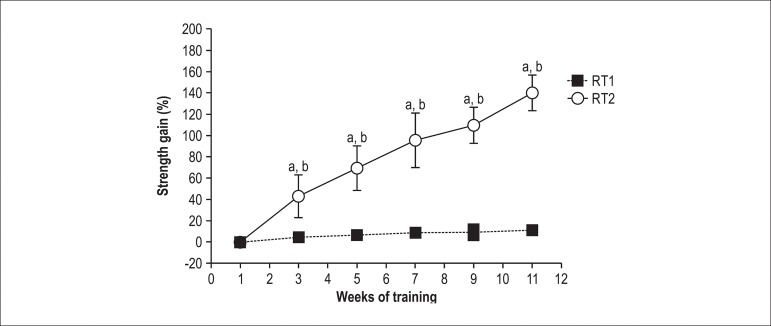
Delta strength gain seen by maximum weight carried in two different protocols for 12 weeks. a, p < 0.05 vs. pre (1^st^ week); b, p < 0.05 VS. RT1. All values are presented as means ± SD.

SHR RT2 group presented a progressive increase in muscle strength compared with the first week (p < 0.05), while the muscle strength of the RT1 group did not increase throughout the intervention (p > 0.05). Considering both training protocols, the RT2 group had a muscle strength gain of 140 ± 16.6%, while the SHR RT1 group increased strength by 11 ± 4.8% (p < 0.05) (Figure 4). Although BW of the hypertensive rats remained unchanged during the study, the RT2 group showed a progressive increase in muscle strength relative to BW (p < 0.05). The RT2 group exhibited a muscle strength gain relative to BW of 118 ± 28.3 %, and this increase was significantly different as compared with the RT1 group in which the increase was of only 0.1 ± 3.4% (p < 0.05).

### Total Overload

**Figure 5:**
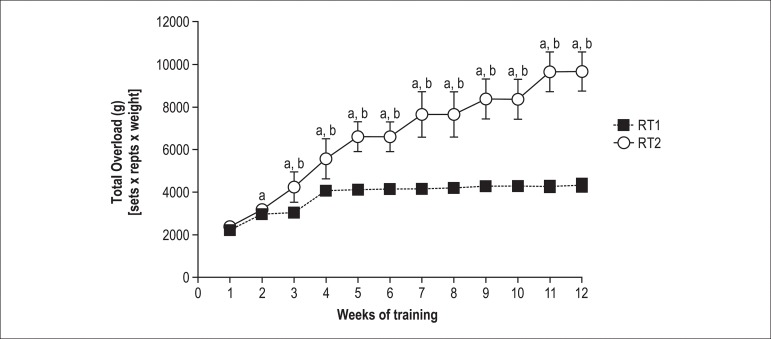
Total overload in grams for the 12 weeks of resistance training. a, p < 0.05 VS. pre (1^st^ week); b, p < 0.05 vs. RT1 group. All values are presented as means ± SD.

The total overload consisted of the sets•repetitions•weight performed throughout the training weeks (i.e., all climbing sets held in the week added), and is presented in Figure 5 for the studied groups. The RT1 group displayed an increase in total load carried from the second week and the remaining weeks as compared to the first week (p < 0.05). The RT2 group also displayed a significant increase in this variable from the second week, and this difference was maintained throughout the experimental protocol compared with the first week (p < 0.05). In the 3^rd^ week of training the RT2 group had a significant increase as compared with the RT1 group, and this pattern was maintained until the 12^th^ week of training (4337 ± 280 vs. 9659 ± 928 g, RT1 and RT2 respectively; p < 0.05).

## Discussion

The effects of the intensity of RT (as % of MWC) on BP and muscle strength of SHR were evaluated. The results indicated that, although the heavier RT protocol had elicited higher muscle strength gains, the chronic benefits of both protocols on controlling BP in animals with SH were similar. While some studies have demonstrated the benefits of AE in untreated severely hypertensive rats^26^ and humans under medication.^11,12^ Moraes et al showed that moderate-intensity RT also reduces BP in non-medicated men with stage 1 hypertension similarly to the AE, and in addition to gains in muscle strength.^6^

To the best of our knowledge, this was the first study analyzing the efficacy of resistance exercise and the role of training intensity for SHR with SH. Other authors, such as Araujo et al.,^27^ already had demonstrated the efficacy of RT on BP control in animals with stage-1 hypertension (drug-induced) trained at moderate-intensity RT (50% of one-repetition maximum for four weeks). In the present study it was possible to prevent the BP increase in SHR undergoing 12 weeks of RT, regardless of the training intensity, suggesting that both intensities of RT protocols (i.e.~40% and 70% MWC) were effective as an antihypertensive nonpharmacological therapy. Furthermore, intensities of approximately 40-70% 1RM are considered suitable as a safe recommendation for hypertensive patients.^4-7^

Maintaining BP levels is very important, since each 10 mmHg increase in BP levels is associated with a 25% increase in the risk of myocardial infarction and stroke.^26^ Furthermore, it has been demonstrated that 12 weeks of RT induce changes in the cardiovascular risk factors, such as decreased lipid content in the liver, mesenteric and retroperitoneal fat depot, blood lipids and atherogenic index in ovariectomized rats.^28^

By the end of the training protocols, RT1 and RT2 groups showed a downward trend on SBP by 5 mmHg and 7 mmHg (p > 0.05), respectively. These results reflect a low cardiac overload demonstrated by the evaluation of the RPP. In contrast, in the SED-SHR group there was a significant increase by 19 mmHg in BP, with a high RPP (p < 0.05). It has been shown that the decrease in HR, as observed in our RT1 group (lower intensity), may be explained by a modulation of baroreflex sensitivity leading to a decreased sympathetic tone.^29^

When we compared our data to those of other studies using AE in SHR also with elevated BP,^16,30,31^ a similar result was found in terms of inhibition of the resting BP elevation throughout the experimental period. In view of these findings, a moderate-intensity RT also appeared to be promising in a severe condition of hypertension. Faria et al.^19^ e Lizardo et al.^20^ found that moderate-intensity acute resistance exercises lower BP and increase the production of nitric oxide in SHR. In this sense, probably this mechanism is involved in the decrease of BP in hypertensive rats.

On the other hand, when a higher intensity was applied (70% MWC), a higher increase of muscle strength (approximately 140% and 118% relative to BW) was elicited for the RT2 group. However, the RT1 group (40% MWC) showed a little but not negligible increase in absolute strength (11%). When these values were adjusted to BW gain in this low power disappeared (0.1%). There is evidence showing that the increase in muscle strength is essential for individuals with hypertension,^10^ probably because of a lower cardiovascular overload presented during activities of the daily living, mainly those in which strength performance is needed, such as carrying shopping bags, climbing stairs or dragging furniture.^9^ Additionally, RT may increase muscle mass, which may be beneficial for the resting metabolic rate, improvement of the immune system, and prevention of falls in the elderly.^5^ Likewise, a recent study conducted, for two decades, in 1,506 men with hypertension suggested that high levels of muscular strength seem to protect these individuals from all-cause mortality.^10^

In our study, the weight of the soleus and gastrocnemius muscles did not increase in the trained groups in comparison to SED-SHR (p > 0.05). Hornberger and Farrar^22^ found the weight of the flexor hallucis longus muscle to be increased after 8 weeks of RT, but not the weight of the soleus, plantar, gastrocnemius and quadriceps muscles. Corroborating our findings, Duncan et al.^32^ also did not find muscle hypertrophy gains in the extensor digitorum longus or soleus muscles after a heavy RT model in Wistar rats. Possibly, both the intensities used, duration of the training, muscles assessed, animal model, and training may explain these distinct results.

### Study limitations

The lack of measurements such as morphological, biochemical and molecular parameters are a limitation of this study, and should be addressed in further investigations. For the present, however, the initial idea was to demonstrate that RT appears to be safe, even in extreme conditions of arterial hypertension. Throughout the training no deaths or incidents with animals were observed. This absence of complications in the study may be linked to the sample size.

## Conclusion

In summary, these findings suggest that different intensities of RT prevent the rise of BP in rats with SH. Moreover, an important result was that the greater-intensity RT induced more expressive gain in muscle strength, without raising the resting BP levels. Thus, RT may function as an adjuvant to pharmacological treatment to prevent BP elevation at rest, in addition to benefiting the muscle strength of hypertensive patients attending a rehabilitation program.
